# Beginning With the End in Mind: Contextual Considerations for Scaling-Out a Community-Based Intervention

**DOI:** 10.3389/fpubh.2018.00357

**Published:** 2018-12-10

**Authors:** Laura E. Balis, Thomas E. Strayer, NithyaPriya Ramalingam, Samantha M. Harden

**Affiliations:** ^1^Department of Human Nutrition, Foods, and Exercise, Virginia Tech, Blacksburg, VA, United States; ^2^Extension, University of Wyoming, Lander, WY, United States; ^3^Translational Biology, Medicine, and Health, Department of Human Nutrition, Foods, and Exercise, Virginia Tech, Blacksburg, VA, United States

**Keywords:** RE-AIM, physical activity, cooperative Extension system, implementation science, translation

## Abstract

**Introduction:** A number of effective physical activity programs for older adults exist, but are not widely delivered within community settings, such as the Cooperative Extension System. The purpose of this paper was to determine if an evidence-based intervention (EBI) developed in one state Extension system could be scaled-out to a new state system.

**Methods and results:** The RE-AIM (reach, effectiveness, adoption, implementation, maintenance) framework was used to guide an iterative evaluation of three translational stages. Stage 1: Before program adoption, Extension health educators were surveyed and interviewed to assess physical activity programming perceptions and factors that may influence their decision to attend training or deliver the program in practice. Results indicated that a virtual, scalable training protocol would be necessary and that training needed to include hands-on instruction and be catered to those who were less confident in physical activity program delivery. Stage 2: Training attendees were surveyed pre- and post-training on factors related to the adoption-decision making process and contacted post-training to assess program delivery status. Training did not influence perceptions of the program, intent to deliver, or confidence in delivering the program. Stage 3: During program implementation, the program was evaluated through the RE-AIM framework by surveying across three key stakeholder groups: (1) program participants, (2) potential delivery personnel, and (3) Extension administrators. Findings indicate that the program has the potential to reach a large and representative proportion of the target audience, especially in rural areas. However, adoption and implementation rates among Extension health educators and community partners were low and data collection for effectiveness, implementation, and maintenance was a challenge.

**Conclusion:** Overall, the results indicate initial struggles to translating and evaluating the program in a large, rural state. Implications for practice include making system-level changes to increase physical activity program adoption rates among Extension health educators and improve data collection and program evaluation through this community-based organization. More work is needed to identify infrastructure support and capacity to scale-out EBIs.

## Introduction

Delivering (and sustaining) evidence-based interventions (EBI) in community practice is challenging. One challenge is that delivery personnel have to select an intervention and ensure its fit within the mission, values, and resources of the system they are delivering (1). County-based delivery personnel in the land-grant university Cooperative Extension Service (Extension) have the autonomy to select open-access (i.e., open to all, without restriction due to sex, socioeconomic status, or, more notably, health condition) interventions for their county residents (2). One target audience for whom Extension professionals provide services is older adults. As only 12% of this population is meeting physical activity recommendations, and new efforts within Extension are geared toward promoting physical activity (3–5), it is necessary to understand existing programs, their impacts, and how to scale-out interventions (6) across Extension state systems. Scale-out is “a deliberate effort to broaden the delivery of an EBI. Scale-out is an extension of scale-up and uniquely refers to the deliberate use of strategies to implement, test, improve, and sustain an EBI as it is delivered to new populations and/or through new delivery systems that differ from those in effectiveness trials.” [(6) p. 3].

One way that Extension has been challenged at scale-out is that rather than scaling core elements of “what works,” new or unique programs are introduced to the system. This is evidenced by a recent systematic review of open-access physical activity interventions for older adults which found that 17 unique open-access physical activity programs were offered by Extension professionals (7). In addition to duplicated efforts, both the fidelity to the underlying evidence-based program principles and the impact of these open-access interventions on older adult physical activity levels is underreported (7).

In order for this process of scale-out to be successful, information about how and why the intervention works is needed, as much as whether the intervention worked or not (6, 8, 9). Notably, there are three types of scale-out: EBI scaled-out to (1) the same population through a different system, (2) different population, same delivery system, and (3) different population and different delivery system (6). However, while the national Extension system is “one delivery system,” the structure of each state may be somewhat unique. In addition, while “older adults” may be one population, older adults from one state may experience different barriers and facilitators to program adherence when compared to older adult population in a different state. Therefore, the degree to which an EBI can be scaled-out from one state Extension system to another is difficult to discern.

One way to understand scale-out of EBI from one state system to another is to use pragmatic data collection. Pragmatism focuses on “issues and data relevant for making decisions and taking action [10, p.257].” This type of evaluation is especially useful in community settings that may not have substantial research funding, and can move beyond evaluating intervention effectiveness and determine for whom, where, when, why, and how an intervention is working in a given context (11). The RE-AIM (reach, efficacy, adoption, implementation, maintenance) framework can be used to systematically capture perceptions, decision making, and impacts (8, 9, 12, 13).

Using these key considerations, researchers and practitioners can iteratively engage to reflect on successes and failures related to adoption and implementation through a participatory approach (1). Collecting pragmatic measures within a participatory approach is crucial to understand how and why an evidence-based intervention may be delivered outside of the context in which it was developed. Taken together, the research question was: Can an EBI developed in one state Extension system be scaled-out to a new state system? Capturing this context-driven work is essential to understanding why and how interventions are adopted, implemented, and maintained within delivery systems so they can be scaled-out to reach broader populations.

## Materials and Methods

### Design

As recently proposed by members of the National Working Group on RE-AIM Planning and Evaluation Framework (14), the RE-AIM framework was applied across three stages of implementation: before adoption, during adoption, and during program implementation; herein called the scaling-out process. First, before the program was adopted, a mixed-methods design based on RE-AIM was used to capture perceptions of Extension health educators who could deliver the program in practice. Second, during program adoption, a pre- and post-training survey based on RE-AIM adoption was used to understand delivery personnel's adoption-decision making process. Finally, as the program was implemented, it was comprehensively evaluated through RE-AIM, including pre/post and cross-sectional measures, depending on outcome.

**Figure 1:**
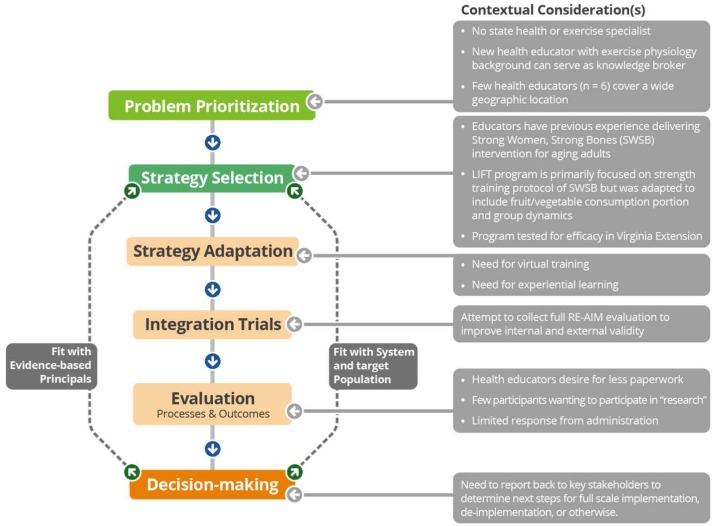
Integrated research-practice partnership model with contextual considerations leading to translational solutions.

In addition to the planning and evaluation framework, an adapted participatory process model was used to guide this work. Figure 1 [adapted from Estabrooks et al. 15] details the contextual considerations that influenced each stage of the process. Notably, there were challenges in visually representing the iterative process of contextual considerations and responses. While the work here is presented in stages, it is important to note that these processes are continuous steps in order to ensure that the researchers, Extension health educators, and community partners engaged in a reflective and action-oriented manner. This study was approved by the University of Wyoming (stage 1) and Virginia Tech (stages 2 and 3) Institutional Review Boards.

### Setting

Wyoming is a large, rural state with a small population (579,315). Ten Extension health educators cover the state's 23 counties and the Wind River Indian Reservation (by stage 2 of this research, only six health educators were employed in the state). These educators have a broad reach, interacting with over 40,000 participants per year. In Wyoming, there is no Extension health or physical activity specialist. When a new county-based Extension health educator (with a background in physical activity promotion and research) was hired by University of Wyoming Extension (UWE), the educator contacted the Virginia Cooperative Extension (VCE) Exercise Specialist to inquire about potential research partnerships. The new county-based Extension health educator served as a knowledge broker (i.e., an intermediary between researchers and stakeholders who facilitates knowledge transfer between these parties) (16). In alignment with the integrated research-practice process (IRPP) model, this setting and work identified that the lack of a health specialist and the small number of Extension health educators covering the state were problems to be addressed in order to successfully implement a physical activity intervention (see Figure 1: problem prioritization).

### Program Development and Description

Lifelong Improvements through Fitness Together (LIFT) (17, 18) was adapted from two programs: Stay Strong Stay Healthy (19) and Activity for the Ages (20). LIFT combines the evidence-based behavioral strategies (goal setting, self-monitoring, and group dynamics) from Activity for the Ages with the evidence-based strength training protocol from Stay Strong Stay Healthy (18) and Strong Women, Strong Bones (also delivered by Extension professionals) (21). Since LIFT was adapted and tested specifically in Virginia, it was unknown whether the packaged LIFT program could be scaled-out to a new Extension state system (see Figure 1: strategy selection).

## Stage 1: Before Adoption

Before offering a LIFT training, the exercise specialist and UWE knowledge broker conducted a pragmatic concurrent, transformative mixed-methods [i.e., equal emphasis on the quantitative and qualitative findings 22] study. To aid in replicability, the survey and interview guide can be found in Appendices A and B. Briefly, 67% of the eligible Extension health educators completed the online survey. Overall, participants were middle-aged Caucasian females; more demographic characteristics are displayed in Table 1. Health educators reported a range of comfort for delivering physical activity (from moderately to very comfortable). While none of the Extension health educators were currently delivering physical activity programs, three (50%) were thinking about offering a physical activity program (contemplation phase) (23). Finally, most of the survey respondents (*n* = 5) indicated that they would be interested in receiving training (either in person or via webinar) on delivery and evaluation of the LIFT program.

**Table 1:** Extension health educator characteristics compared to LIFT training participant characteristics.

**Demographics**	**Extension health educators (*N* = 6)**	**LIFT training participants (*N* = 7)**
	**Mean (**±**SD)**	**Mean (**±**SD)**
Age	50 (±17.8)	46 (±15.0)
	**N (%)**	**N (%)**
**GENDER**		
Female	6 (100)	5 (71)
Male	0 (0)	2 (29)
**RACE**		
White	5 (83)	5 (71)
Other race	1 (17)	2 (29)
**ETHNICITY**		
Non-Hispanic	4 (67)	7 (100)
Hispanic	0 (0)	0 (0)
Not sure	2 (33)	0 (0)
**EDUCATION LEVEL**		
Some college	0 (0)	4 (57)
Bachelor's degree	2 (33)	1 (14)
Graduate degree	4 (67)	2 (29)
**HEALTH SELF-RATING**		
Excellent	3 (50)	2 (29)
Very good	2 (33)	1 (14)
Good	1 (17)	2 (29)
Fair	0 (0)	2 (29)

Thematic coding of the two interviews yielded 349 meaning units reporting perceived barriers (*n* = 115 meaning units) and facilitators (*n* = 157) for delivering physical activity programs and types of programs delivered (*n* = 71). See Table 2 for coding, frequencies, and example meaning units. For barriers, interviewees expressed that their workload was too high to incorporate additional types of programming and their focus was on nutrition. They also mentioned that delivering the Strong Women, Strong Bones program (which was previously implemented through UWE) was too time consuming. As for facilitators, interviewees had positive perceptions of physical activity recommendations and benefits and had enjoyed the Strong Women, Strong Bones training. Community partners were mentioned as a source of physical activity program delivery. While a lack of facilities came up as a barrier, the presence of facilities (e.g., schools, fairgrounds, and walking paths) was also mentioned as a facilitator. Finally, regarding types of programs delivered, interviewees indicated that they were not currently delivering any physical activity programs. Rather, physical activity was promoted through incorporating topics, such as “sit less, move more” in other programming.

**Table 2:** Qualitative results of Extension health educators' perceptions of physical activity programs (*n* = 2 interviewees).

**Theme**	**Subtheme (*n* = meaning units)**	**Category (*n* = meaning units)**	**Example meaning unit**
Perceived barriers to delivering physical activity programs	Educator personal factors (*n* = 51)	Workload (*n* = 17)	And we never can say, “oh If I add this I can let that other thing go so pretty soon it's like so much that that you don't do the new stuff [programming].”
		Difficulty meeting physical activity recommendations (*n* = 12) Strength training (*n* = 7) physical activity (*n* = 5)	And so I think again you'd just have to ahh try to work that [strength training] into your routine every day and I don't.
		Lack of confidence (*n* = 9)	Because you don't feel as comfortable yet so you stick with the old stuff [programming].
		Lack of communication with colleagues (*n* = 6) Out of state colleagues (*n* = 3) Local colleagues (*n* = 3)	That I don't know. I don't have any, I guess I don't have any idea on what's going on elsewhere [physical activity programming in other states].
		Lack of interest in training (*n* = 5)	Personally, no [I wouldn't be interested in a training or certification for physical activity].
		Other influential characteristics (*n* = 3) Age (*n* = 1) Lack of training (*n* = 1) Unfamiliar with facilities (*n* = 1)	Well I think probably my biggest barrier is I'm getting old.
	Organizational structure (*n* = 27)	Job focus area (*n* = 14)	I have to concentrate a lot on um cooking and nutrition through cooking and that's sort of just where I went.
		Lack of organizational support (*n* = 6)	So I do know…. we don't think that's [delivering Strong Bones] quite the best use of our time.
		Multiple areas served (*n* = 5)	So there's five counties [that I'm employed in].
		Single-county programming (*n* = 3)	But I do a lot of little individual programing within my area too.
	Program factors (*n* = 25)	Time required (*n* = 10)	For that one [Strong Bones]… it's time consuming you know.
		Heavy weights required (*n* = 4)	Another thing was we had hand held weights and carrying those little guys around I mean they're heavy.
		Costs (*n* = 3)	…because of how much it costs you know for the gas because when we go to other counties the university reimburses us for fuel and that was you know a pretty big commitment the 2 days a week as far as financial.
		Travel (*n* = 3)	That's a tough life you know to keep going back and forth.
		Community partners not available to deliver (*n* = 2)	I just couldn't seem to find a volunteer and the senior center didn't have enough staff to commit to it [delivering Strong Bones].
		Participant recruitment (*n* = 2)	And so once you kind of do something you have to not do it for a while because probably everyone that was interested did it the first go round so you need to kind of wait and a few years and get a new bunch of people that might be interested.
		Lack of variety (*n* = 1)	The one thing I know by the end of the 8 weeks the people were getting tired because they were of the same exercises, it was the exact, you know it would be like doing an aerobics class with the exact same routine for 8 weeks. And so with that one you know I don't think we changed.
		Maintenance of physical activity (*n* = 1)	Whether they stick with it or not is you know is another issue.
	Facilities (*n* = 10)	Lack of facilities (*n* = 4)	We don't have like a YMCA or a rec center.
		Lack of space (*n* = 3)	But you know that was when I did it [Strong Bones] … I had in the building I have we have a conference room so you'd have to move all the tables and chairs and then put them all back.
		Facility operating hours (*n* = 3)	The weight room we have a great facility it's just such limited hours.
	Weather (*n* = 2)	Winter weather (*n* = 1)	But I think one of our biggest barriers that we face is um our weather is not conducive to um outdoor stuff. In the winter it's cold and snowy and icy and the wind is blowing. So if you don't have a place to do that inside it makes it really hard. And winter, I mean a lot of times by the first of October till may were in winter, you know.
		General weather (*n* = 1)	But I think weather is a huge thing for us.
Perceived facilitators to delivering physical activity programs	Educator personal factors (*n* = 61)	Positive perception of physical activity (*n* = 22) Aerobic activity recommendation (*n* = 7) Active lifestyle (*n* = 5) Belief that older adults need physical activity (*n* = 4) Need for strength training (*n* = 2) Strength training (*n* = 2) Health benefits (*n* = 1) Ease of meeting recommendations (*n* = 1)	I've always just believed it's really good for your health.
		Types of activities used to meet physical activity recommendations (*n* = 8) Walking (*n* = 5) Exercise bike (*n* = 2) Gym (*n* = 1)	Well I just try to set time, 5 days a week, you know I exercise, um my walking partner has just started going south for the winter, but we would usually do like 2 days in the gym.
		Positive perception of physical activity programming (*n* = 7)	We've always felt it's important and if we can speak about it and add a little tidbit here and there that's good so I think we're coming along with having an actually subcommittee that's maybe gonna bring forth some programs or help strengthen that area [physical activity] you know by whichever tactic they take.
		Strategies used to meet physical activity recommendations (*n* = 6) Fitbit for motivation (*n* = 3) Habit (*n* = 1) Working around weather (*n* = 1) Creativity (*n* = 1)	I also have like a Garmin, Fitbit whatever you want to call it, so that helpful, reminds me to move, it reminds me to you know how far I have walked, so.
		Peer influence (*n* = 4)	So I think they [younger educators] are all very excited and they're trying to get us older ones on board, so.
		Positive perception of Strong Bones program (*n* = 3)	Um, You know that little handheld weight program with um, Strong Women was easy.
		Confidence (*n* = 2)	Um I would say, like do you want a rating scale, I'd say fairly confident [in successfully delivering physical activity programming].
		Knowledge of physical activity recommendations (*n* = 1)	I know kids have a different recommendation.
	Organizational factors (*n* = 38)	Initiative team (*n* = 11) Need team buy-in (*n* = 6) Programs should be chosen as a team (*n* = 3) Willing to deliver programs chosen by team (*n* = 2)	Because if you don't, there's so few of us, there's what like 10 if we're fully staffed, and I think we're down to seven maybe or eight now, and so there's so few of us so in order to get the kind of impacts you need to all buy into the same thing.
		Issue team (*n* = 7) New active living team (*n* = 4) Issue team structure (*n* = 3)	And for the first time we actually have an issue team that deals with physical activity.
		Job performance evaluation (*n* = 6)	But um they [superiors] you know want us to be out teaching and educating.
		Organizational support (*n* = 4)	We have a very progressive person as our nutrition and food safety administrator and I think she would be really behind this, so.
		Organizational change (*n* = 3)	And so whoever takes over my position will be 100% nutrition and food safety.
		Role in Extension (*n* = 2)	I am a nutrition and food safety educator.
		Age difference (*n* = 2)	I think the young ones, we're kind of divided right now, there's three of us that I would consider in the older group (laughing) and the rest of them are all very young.
		Educators value physical activity (*n* = 2)	You know I think we all [Wyoming Extension health educators] realize physical activity is very important.
		Need for program evaluation (*n* = 1)	And then you know we'll have our evaluations ready and go from there.
	Physical activity training (*n* = 31)	Past training (*n* = 16) Training components (*n* = 9) Programs trained on (*n* = 5) Perceptions of training (*n* = 2)	[I enjoyed Strong Bones training] cause there were some of the exercises I wasn't sure if I was doing them exactly right or not you know.
		Desire for training (*n* = 11) Desired training components (*n* = 5) Belief in importance of training (*n* = 3) Program type (*n* = 2)Need for resources (*n* = 1)	Maybe having to um do some of the program in front of some of the rest of us. Um hands on as far as that goes.
		Current training methods (*n* = 4)	So basically what I keep up with is you know if I find a webinar that's interesting.
	Community partners (*n* = 14)	Delivery support (*n* = 11)	That we could work with someone in our community and get that [physical activity program] delivered.
		Partnerships with local organizations (*n* = 3)	Yes. Yes, I do [generally have partnerships with the senior center].
	Facilities (*n* = 9)	School (*n* = 3)	Um, I think that's one thing, our school makes it easy, like, we can use their hallways, and we try to do things sometimes in conjunction with the schools.
		Fairground (*n* = 2)	And we try to have things here at our fair grounds, for like if we have a 4-H event we have a basketball hoop here and we're gonna install horseshoe ends, and so we are trying to do some things that encourage physical activity.
		Walking path (*n* = 2)	Having a place to do it um you know we have some bike paths well we have a walking bike path which is good.
		Playground (*n* = 1)	And we do have really nice playgrounds in town, so it's easy to like have a 4-H activity maybe at the park where the kids can use the equipment that we have.
		Pool (*n* = 1)	Um, we just got, one thing is we just got a brand new swimming pool so the kids are excited, we have tried to incorporate going to the pool into our 4-H activities.
	Program factors (*n* = 4)	Resources available (*n* = 2)	And some people would have their own weights and some wouldn't so that's why we always had weights with us that we carried so that if someone didn't have their own we would provide them with some if I can remember them right the weights were from like one or two pounds up to maybe 10 just those little hand held dumbbells.
		Target audience interest (*n* = 2)	And every year we have to reprint those [walking program] maps for everyone to use ‘cause they like them so much, so that's been kind of a cool thing we did.
Types of programs delivered	Physical activity programs delivered (*n* = 32)	Past programs delivered (*n* = 18)	We've [in the past] done things like have a treasure hunt where they [4-H kids] have to follow a treasure map and count their steps and find the treasure.
		Program adaptation (*n* = 11)	And then probably have to make some adjustments. Because even if it is a program from another state, we like to “Wyomingize” it make it for our clientele so it's successful.
		Needs assessment methods (*n* = 3)	Um we have a focus group (pause) system of um clientele assessment that we use um we meet in different counties every year and then we get the various county reports plus a statewide summary we use.
	Methods of promoting physical activity (*n* = 17)	Dissemination methods (*n* = 9)	So I tried to get the word out [on physical activity] vs. doing it with them in a class.
		Physical activity topics delivered (*n* = 8)	I mean with my, I do a variety of projects so for example at the senior center my last topic was sit less move more.
	Program target audience (*n* = 11)	Older adults (*n* = 4)	Uh elderly I'm guessing they [Strong Bones participants] were oh probably 60 and up.
		Adults (*n* = 3)	We've had anyone from 20 to probably 75 [in Dining with Diabetes].
		Diabetics and their caretakers (*n* = 3)	And some of them are like the wife of someone with diabetes.
		All age groups (*n* = 1)	Um my primary I don't know I do everything from youth through seniors.
	Nutrition/food safety programs delivered (*n* = 6)	Current programs delivered (*n* = 3)	Um just trying to think what else we've done recently. I do a lot of food preservation, but there's really no physical activity portion to that.
		Past programs delivered (*n* = 3)	Um we've done Dining with Diabetes here numerous times here.
	Programs not delivered (*n* = 5)	Not currently doing physical activity programs (*n* = 4)	I think the lack of not having anything real formal since the Strong Women, you know the Body Works program, we haven't really had anything formal since that, it's been quite a few years, maybe even more 3 years.
		Not currently doing physical activity in nutrition programs (*n* = 1)	Um it you know I do um nothing specific [nutrition programs that include physical activity] at the moment.

## Stage 2: During Adoption (Training)

Based on the results of Stage 1, the research team decided that there was a need for a scalable training protocol (i.e., one that was feasible across the entire 97,818 square miles of Wyoming) designed to target Extension health educators who were less confident in delivering physical activity programs and include hands-on instruction and teach-back. LIFT training was created as a 4 h “live” virtual format based on evidence-based methods on training (24, 25), learner-centered teaching (26), and program adoption rates (27). Training included detailed descriptions of program principles and opportunities for experiential learning (e.g., practicing and receiving feedback on the exercises and fitness assessments). Additionally, the training was made available to Extension health educators' community partners (e.g., staff from senior centers and other health organizations) to promote a delivery model that would address the time requirement barrier; Extension health educators were encouraged to attend training with their community partners so they could offer support with implementation and evaluation. See Figure 1: strategy adaptation.

In alignment with Extension practices, pragmatic recruitment and feedback methods (e.g., listserv email invites and post-training surveys) were used. Nine participants completed the initial LIFT training in September 2017: the knowledge broker, one Extension health educator, and seven community partners (including one retired Extension health educator). Following this training, additional community partners expressed interest in the training, and the research team offered another training in December 2017 to four community partners.

Of the thirteen total training participants, nine (69%) completed surveys. Of the seven who completed pre-training surveys, trainees were predominantly middle-aged Caucasian females. All participants (100%) were very or completely confident in meeting physical activity recommendations. While all participants (100%) reported high physical activity levels through the International Physical Activity Questionnaire (IPAQ), only 57% reported meeting strength training guidelines. Pre-training, participants agreed that they intended to deliver LIFT and to include LIFT in a plan of work; mean values were both 4.00 (+0.707). The Wilcoxon signed-rank test showed that post-training surveys did not detect a statistically significant change in intent to deliver LIFT (*Z* = 0.000, *p* = 1.000) or inclusion of LIFT in a plan of work (*Z* = −0.447, *p* = 0.655).

The program characteristic that influences the adoption-decision making process of highest importance was “The program has been successful when tested in community settings” (mean rating of 4.00 = very important). While the majority of the factors (73%) had a mean rating between 3.00 and 3.99 (moderately important), four factors rated as only “somewhat important”: “I do not feel that the program is part of my job responsibility,” “I do not feel comfortable delivering the program,” “I am not physically active, so do not feel comfortable delivering a physical activity program,” and “I do not have the expertise that is needed to deliver the program.” By February 2018 (5 months after the first training and 2 months after the second), three LIFT programs were being delivered through UWE, and the comprehensive evaluation was initiated. Due to this low initial implementation rate, a survey for trainees who both did and did not implement the program was added to the measures for adoption.

## Stage 3: During Implementation

### Participants and Recruitment

The final step in this research was to evaluate both the individual-level impact and the system-level delivery of LIFT through the RE-AIM framework. The evaluation included three levels of respondents in an attempt to collect data on all RE-AIM dimensions: *reach* and *effectiveness* (LIFT participants), *adoption* and *implementation* (delivery personnel, i.e., both Extension health educators and community partners), and system-level *maintenance* (UWE administrators). LIFT participants were recruited through senior centers (including participants in Strong Bones programs), newspaper articles, flyers, and word of mouth. Extension health educators and community partners were contacted via email after the LIFT training to complete a brief online survey. Extension administrators (*N* = 3) were also contacted via email to complete a brief online survey.

### Data Collection and Analysis

Data were collected on all RE-AIM dimensions except for individual-level maintenance, which was outside the scope of this work (see Figure 1: integration trials). Measures for each dimension were as follows (see Table 3 for detailed aims and outcome measures):

**Table 3:** RE-AIM dimensions and measures.

**Dimension**	**Aims and outcome measures**
Reach: Number, proportion, and representativeness of LIFT older adult participants	**Aim:** To monitor and evaluate older adult participation rate **Outcome Measure:** Number of LIFT participants, demographic items through pre-program survey
Effectiveness: Impact on primary outcomes, quality of life, and unintended consequences	**Aim:** To confirm the effectiveness of LIFT at improving functional fitness and increasing physical activity levels **Outcome Measure:** Functional Fitness Assessments, International Physical Activity Questionnaire through pre- and post-program surveys
Adoption: Number, proportion, and representativeness of settings and staff who deliver the intervention	**Aim:** To monitor and evaluate Extension health educator and community partner adoption rate; to understand factors influencing adoption **Outcome Measure:** Number of Extension health educators and community partners implementing LIFT, demographic items through pre-training survey; acceptability, appropriateness, and feasibility of LIFT through follow-up survey
Implementation: Degree to which intervention was delivered as intended	**Aim:** To determine the degree of fidelity to which LIFT is delivered by Extension health educators and community partners **Outcome Measure:** Process evaluations
Maintenance (system level): Extent to which delivery/ implementation is sustained over time	**Aim:** To evaluate administrator support of LIFT **Outcome Measure:** Acceptability, appropriateness, and feasibility of LIFT through follow-up survey

#### Reach

LIFT participants completed baseline surveys including demographic items used to calculate reach (proportion and representativeness).

#### Effectiveness

The pre- and post-program surveys included IPAQ items to assess whether participants were meeting physical activity recommendations (28). A validated seven-item test associated with performing everyday activities independently (29) was completed at baseline and post-program, as an additional outcome of LIFT is improving functional fitness.

#### Adoption

The primary adoption indicator was the total number and representativeness of those trained on LIFT program (including both Extension health educators and community partners). In addition, all those eligible to deliver the LIFT program were asked to complete a survey assessing: (1) acceptability, appropriateness, and feasibility (30) of implementing LIFT (on a 5-point Likert scale; 1-completely disagree, 5-completely agree), and (2) their current stage of change category (23) based on the 6-point scale of 1- “I am not considering delivering LIFT in my county at all” to 6- “I have been delivering LIFT for 6 months of more.” Demographic items were not included to create a short survey that decreased respondent burden.

#### Implementation

This was assessed through process evaluations designed for delivery personnel to self-report the extent to which the program was delivered as intended and capture adaptations made during program delivery. The process evaluations contained five categories: warm-up activity, group-dynamics strategy, exercises, cool down, and overall program delivery.

#### Maintenance

As a proxy measures for system-level maintenance, administrator perceptions were sought related to: (1) acceptability, appropriateness, and feasibility (30) of LIFT; (2) the importance of RE-AIM factors for LIFT [e.g., “The program has potential to attract/recruit a group of participants that is representative of the residents of Wyoming (reach); LIFT has been previously tested in community settings (effectiveness): 1-not at all important, 5-very important], and (3) whether they supported educators in delivering LIFT (yes or no with reasons why or why not). Demographic items were also not included in this survey.

Means and standard deviations of continuous variables and frequencies and proportions of nominal variables were calculated for the overall sample. Representativeness was calculated by comparing demographics (age, gender, race, ethnicity, education level, and work status) of LIFT participants to all older adults (age 65 and older) in Wyoming (city or county level census data was not available) (31). As raw data was not available for education level or work status, frequencies were calculated by using census data percentages and totals. Representativeness of BMI was calculated by comparing LIFT participants to the sub-sample of older adults in Wyoming (age 65 and older) who were selected and responded to the Behavioral Risk Factor Surveillance System (BRFSS) survey. A one-sample *t*-test was used to compare mean age; Fisher's exact test was used to compare categorical variables due to the small sample size.

### Results

#### Reach

Forty-eight participants attended the LIFT classes. However, only 37 individuals agreed to the research portion of this work. Of the 37 who agreed to be research participants, 18 completed pre-program surveys. These participants had a mean (±SD) age of 67.8 (±4.9) years, were predominantly retired (78%), and were Caucasian females (100%). Participants had a mean (±SD) BMI of 29.9 (±*7*.0) with seven (39%) classified as obese, seven (39%) classified as overweight, and four (22%) classified as normal weight. For each of the three delivery locations, proportion of LIFT participants was calculated as 17 out of 1,480 adults age 65 or older (1%) in Lander, 12 out of 35 (34%) in Pavillion, and 8 out of 281 (3%) in Guernsey. When comparing the representativeness of LIFT participants to older adults in Wyoming, there were no significant differences in terms of race (*p* = 1.000), ethnicity (*p* = 1.000), employment status (*p* = 1.000), education level (*p* = 0.297), and BMI (*p* = 0.324). There was a significant difference in age and gender: LIFT participants were younger (*t* = −4.385, *p* = 0.000) and more likely to be female (*p* = 0.000) (see Table 4).

**Table 4:** LIFT participant characteristics compared to older adults (age 65+) in Wyoming.

**Demographics**	**LIFT Participants (N = 18)**	**Older Adults in Wyoming**
	**Mean (**±**SD)**	**Mean**
Age	67.8 (±4.9)	73
	**N (%)**	**N (%)**
**GENDER**		
Female	18 (100)	45,921 (52)
Male	0 (0)	41,891 (48)
**RACE**		
White	18 (100)	84,488 (96)
Other race	0 (0)	3,324 (4)
**ETHNICITY**		
Non-Hispanic	18 (100)	83,649 (95)
Hispanic	0 (0)	4,163 (5)
**EDUCATION LEVEL**		
High school graduate or some college	11 (61)	51,814 (64)
Bachelor's degree or higher	7 (39)	19, 854 (25)
**WORK STATUS**		
Not in the labor force (retired, disabled/unable to work, or homemaker)	16 (89)	64,001 (79)
Employed	2 (11)	16,222 (20)
**BMI**		
Overweight or obese	14 (78)	1,077 (64)
Normal weight	4 (22)	603 (36)

#### Effectiveness

Of the 37 LIFT research participants, 10 completed both pre- and post-program functional fitness assessments. There was a statistically significant increase in the 30 s chair stand test (*t* = −2.673, *p* = 0.028) and no significant difference in any of the other tests (balance station, 30 s arm curl, 2 min step test, chair sit and reach, back scratch, and eight food up and go). As only five participants completed both pre- and post-program surveys, changes in physical activity levels were not included in this report.

#### Adoption

Proportion of delivery agents was calculated for both Extension health educators and community partners. Of the six Extension health educators employed at the time who were invited to the training, one delivered LIFT for an adoption rate of 17%. Of the two Extension health educators who attended training, one delivered LIFT for an adoption rate of 50%. However, the other Extension health educator who attended training indicated through the follow-up email 2 months post-training that she was planning on delivering LIFT but had not yet scheduled a session; she also recruited community partners to attend the second LIFT training and assisted one of the community partners with completing evaluations when she delivered the program.

Of the eleven community partners who attended training, two delivered LIFT for an adoption rate of 18%. Representativeness of those who delivered LIFT compared to those who attended the training but did not deliver LIFT was not calculated, as demographic data from the pre-training survey was only available for one of the educators who delivered LIFT. Demographics of those invited to attend LIFT training (the six Extension health educators) were compared to the demographics of those who attended LIFT training (both Extension health educators and community partners) and completed the pre-training survey (see Table 1). Due to the small sample sizes, representativeness was not calculated.

One of the 13 training attendees and none of the four Extension health educators who did not attend training completed follow-up surveys. Due to the low response rate, survey results are not included.

#### Implementation

One of the two eligible delivery personnel completed process evaluations. One other educator completed the process evaluations, but these data were not eligible for inclusion in analysis as she was also the knowledge broker (i.e., a research team member with the potential to bias responses) (32). Results from the one educator indicated that the program was overall delivered as intended 100% of the time, although adaptations were made to program components: the warm-up was delivered with 63% fidelity, group dynamics-based activities 75%, strength training exercises 100%, and cool-down 69%. Due to the small sample size, these data should be interpreted with caution.

#### Maintenance

One of the three administrators completed the surveys; due to the low response rate, administrator survey results are also not included.

### Discussion

Results of this work indicated initial struggles in scaling-out a previously tested Extension EBI to Wyoming Extension. All three stages were equally important in capturing the challenges and facilitators of the LIFT EBI scale-out; however, a lack of compliance with data completion (see Figure 1: evaluation processes and outcomes) highlights challenges of a pragmatic approach to data collection (e.g., without large funds for systematic evaluation). This is notable as the researchers were also from within the Extension system, and therefore, aligned evaluation with standards of practice. Therefore, translational solutions (Figure 1) are yet to be determined. However, there were notable observations and implications, by stage, to be shared.

#### Stage 1: Before Adoption

In order to improve physical activity program adoption rates among Extension health educators, system-wide changes are needed. While it appears that the UWE organizational structure supports physical activity programming (e.g., interviewees mentioned a newly developed Active Living issue team tasked with choosing physical activity programs to deliver), educators face barriers to adopting these programs, including role clarity, traditional delivery models, and organizational culture.

Physical activity is not explicitly included as a focus area of Extension health educators' work. Including “physical activity promotion” in Extension health educators' job descriptions and changing position titles to be more inclusive of physical activity could help educators prioritize physical activity programming and de-implement other work duties that are not evidence-based (33). In addition, physical activity programming is fairly new as an Extension target area and Extension educators (in both health and other disciplines) not aware of this change may not support physical activity programming among their colleagues. Interviewees also mentioned a lack of communication with colleagues, both those on their initiative team as well as educators in neighboring states, which makes program dissemination difficult (34). Future work should investigate the usefulness of a dissemination network of Extension state specialists that assists educators in staying informed of evidence-based physical activity promotion efforts taking place nationwide (34).

In addition to role clarity within the system, Extension professionals need guidance on how to leverage volunteers and community members to engage participants across a disperse region. That is, novel approaches for program delivery may be necessary to increase the penetration of an intervention across a state system, particularly a large rural one. For example, interview respondents mentioned time required for program delivery as a barrier to Strong Women, Strong Bones (and LIFT requires a similar time commitment); adaptations to the delivery model may encourage adoption. They also mentioned a lack of funds to cover fuel to reach geographically disparate communities. A train-the-trainer delivery model may help Extension health educators to adopt the program and then turn it over to community partners, as has been done by Washburn and colleagues for their version of LIFT (35). To address lack of time as a barrier, Extension health educators also need training on evidence-based programming; this training could encourage them to adopt existing structured, evidence-based programs instead of developing their own programming (36). Future studies should explore the effects on program adoption rates when these system-level changes are made.

Finally, the culture of the organization and the state can also affect physical activity program adoption. Wyoming is a politically conservative state with an individualistic and independent culture. This culture can impede health promotion efforts, as observed in the tobacco control efforts of the 2000s: “We are independent, we're rugged, we'll smoke if we want to, and do not want any government folks trying to tell us how to live healthier and live longer” (37). This belief can be a barrier to adoption of any program with health behavior change as an outcome. To encourage adoption of physical activity interventions, it may be necessary to shift this mindset within the system.

#### Stage 2: During Adoption

Although perceptions of the LIFT training were positive, participating in training did not change attendees' predictors of implementation, stage of change, or positive intent to deliver both the LIFT program and physical activity programming in general. This may be because those who attended already planned on delivering LIFT (e.g., intent to incorporate physical activity into existing programming and intent to deliver LIFT both had a mean rating of 4 (agree) both before and after training). As these are the top predictors of program implementation, it was expected that program implementation rates following training would be high. However, only 2 months had passed since the second LIFT training, so it is possible that those who attended training will deliver LIFT once they have determined a location, community partners, etc., as intent to deliver LIFT was high post-training. The optimal length of time post-training to assess implementation status is unknown; other studies of Extension-delivery physical activity programs have assessed delivery status after 1 year (Ramalingam et al., in preparation) or annually for 5 years (38). Overall, through this iterative work, attempts were made to include perceptions of delivery agents to “begin with the end in mind (39)”; however, in this study, these efforts to align the intervention with the pull of the system (40) did not lead to strong adoption or implementation. More work is needed to determine what factors would lead to higher implementation rates.

More work is needed to understand the adoption-implementation gap among community partners that occurred following the LIFT training. Training attendees had positive perceptions of the training and intentions to deliver LIFT post-training. Without responses to the follow-up survey, it is difficult to understand perceptions of the program or implementation barriers that occurred. Future research on system-level changes to promote physical activity programming through Extension, training on evidence-based programming for Extension health educators, physical activity program delivery methods that decrease educator time commitment, and barriers to community partner program implementation are needed to address low adoption and implementation rates.

#### Stage 3: During Implementation

Extension is an open-access entity that values pragmatic outcomes; stringent collection of empirical data is more novel to this system and its personnel. Therefore, the magnitude of effect and fit of LIFT within the system is yet to be determined. Furthermore, it was difficult to determine if the program was implemented with fidelity; changes are needed to encourage or incentivize collecting these data from delivery personnel (41, 42). While self-report process evaluations seem easy and manageable, observations may be necessary to monitor program delivery. However, these observations require intense resources (travel, time), so the longevity of this approach may not be feasible. Future work is needed to determine how to train for and monitor high quality delivery.

Although effectiveness, adoption, and implementation data were challenging to collect, there were positive outcomes in terms of reach. First, LIFT demonstrated a strong reach among eligible older adults, particularly in small communities (e.g., 34% of older adults (12 participants) from one community participated). In terms of representativeness, LIFT participants were more likely to be female; however, this is also not surprising as the Wyoming Extension system had previously delivered Strong Women, Strong Bones (21). The program was eventually called “Strong Bones” to be more inclusive but more females participated. One advantage of LIFT is that it is available and promoted to both men and women, similar to the Stay Strong, Stay Healthy program of Extension in Missouri and, more recently, Kansas (19, 43). Although previous research demonstrated that older adults prefer to exercise with other older adults (44, 45), new research shows that gender-segregated classes do not produce better adherence or physical activity outcomes when compared to classes of similar age but mixed-gender (46). From a practical perspective, some older adults may prefer gender segregated classes (44), but LIFT will continue to be offered to all aging adults due to its open-access policy (7).

#### Limitations

The most prominent limitation of this work is the small sample size for empirical data. However, in pragmatic settings large sample sizes are not always available; the purpose of this work was to report process and outcome data in order to aid in replicability and understanding the process of scaling an intervention that was adapted specifically for Extension. Due to low survey response rates, perceptions of LIFT that influence adoption and system level maintenance were not captured. These data may have offered insight into reasons for LIFT not being adopted and implemented (e.g., low perceived acceptability, appropriateness, or feasibility among community partners, Extension health educators, or UWE administrators) and predicted institutionalization of the program. Future work should consider other methods of collecting data to determine perceptions of LIFT and potentially adapt the program to improve adoption and implementation rates. The sample sizes for each portion of this study were small as they were limited by the organization structure of UWE (i.e., only three administrators and six Extension health educators serve the entire state). However, these educators have a large reach, as they cover the entire state and are tasked with providing community-based education to all Wyomingites.

Finally, strategies to partner with delivery personnel could have been better used to enhance buy-in and ensure a good fit between LIFT and UWE as a delivery system (47). The research team did not fully employ an IRPP in UWE, as the LIFT program was developed through an IRPP in VCE. As there were challenges with translating LIFT from Virginia to Wyoming, in the future employing a new, state-specific IRPP is recommended to address potential program adaptations and enhance program sustainability.

This is the first study to follow the scaling-out of an Extension intervention for Extension professionals by Extension professionals that did not include place-based adaptations. For example, Sequin et al. used Extension as a dissemination model for the Strong Women, Strong Bones intervention but were not Extension professionals themselves (48). In another example, a statewide walking program was translated to a new state but the state made place-based adaptations before launching the intervention (1, 49). Reports like this one are needed to show the challenges, successes, and next steps to translating evidence-based interventions across state lines within the national system as well as to other community-based entities that partner with Extension.

## Conclusion

Applying a planning and evaluation framework (e.g., RE-AIM) has the potential to improve transparency and translation of best practices into community settings. However, many settings do not have the resources to capture these iterative, pragmatic data. The results of this study suggest that system-level changes are needed to increase physical activity program adoption rates among Extension health educators, reduce system-level barriers (e.g., role clarity, lack of time or transportation funds), and leverage partnerships to ensure programs can reach those most in need of intervention. Collecting ongoing effectiveness data will be a challenge, and pragmatic ways to indicate a public health impact need to be developed (and match systems' capacity, interest, and value). These improvements in community-based and community-driven data collection may improve reach, effectiveness, adoption, implementation, and maintenance of interventions within Extension.

## Availability of Data and Materials

The datasets used and/or analyzed during the current study are available from the corresponding author on reasonable request.

## Author Contributions

LB and SH conceived of the study, and participated in its design and coordination. LB led the manuscript preparation. NR contributed to data collection and analysis. TS contributed to data analysis. All authors read, contributed to, and approved the final manuscript.

### Conflict of Interest Statement

The authors declare that the research was conducted in the absence of any commercial or financial relationships that could be construed as a potential conflict of interest.
