# Supplementary material for: Beginning With the End in Mind: Contextual Considerations for Scaling-Out a Community-Based Intervention
Source: Front Public Health. 2018 Dec 10;6:357. doi: 10.3389/fpubh.2018.00357 (PMC6296236; doi:10.3389/fpubh.2018.00357)
Supplement: Supplementary file 1 [file Image_1.pdf]

Appendix A:
Educator Perceptions of Physical Activity Programming in University of Wyoming Extension

The overall purpose of this study is to examine and inform physical activity programming within University of Wyoming Extension (UWE).

Specifically, the purpose of this survey is to determine 1) current physical activity topics and programming within UWE; 2) level of intent to deliver statewide programs in 2016; and 3) the characteristics of the agents who may ultimately deliver physical activity programming across the state of Wyoming. Related to the third aim, the research team would like to gather information about you as an individual; both demographics as well as information about your own engagement in physical activity.

All of the results will be reported as group data, whereby no individual will be identifiable in the results or any reports of the study findings. Any information you provide will remain anonymous. Please answer each question to the best of your knowledge. If you choose not to answer any question, just leave it blank and move on to the next question.

Any questions or concerns about completing this survey can be directed to:

Principal Investigators	Coordinator, Institutional Review Board
Laura Balis<br>Email: lbalis@uwyo.edu<br>Phone: (307) 332-2363<br>130 Eugene Street<br>Lander, WY 82520<br><br>Dr. Samantha Harden<br>Email: harden.samantha@vt.edu<br>Phone: (540) 231-9960<br>1981 Kraft Dr<br>Room 1009<br>Blacksburg, VA 24060	Colette Kuhfuss<br>IRB/IACUC Coordinator<br>Email: ckuhfuss@uwyo.edu<br>Phone: 307-766-5322<br>Office of Research and Economic Development<br>University of Wyoming

PART A: Physical Activity and Nutrition Short Questions

1. How many years have you worked for Extension? ______

2. How many years have you worked at the University of Wyoming Extension? ______

3. What is your highest level of education? ____

4. In what discipline is your Master's degree? _____ ____________________________

5. What is your level of comfort delivering nutrition education programs?

Very Uncomfortable				Very Comfortable
1	2	3	4	5

6. What nutrition education programs are you currently delivering?

7. What physical activity education programs are you currently delivering?

8. Which of the following best describes your current situation?

- ☐ I am not considering offering a physical activity program in my counties at all.
- ☐ I am thinking about delivering a physical activity program.
- ☐ I am preparing to deliver a physical activity program (e.g. have compiled some resources, looked at possible programs/curricula, etc.) in the next 6 months.
- ☐ I am delivering a physical activity program.
- ☐ I continuously deliver physical activity programs.

PART B:

1. How much time do you spend, on average per week, on the following tasks.

Task	Average Time (per week)
Recruiting participants	
Tailoring program materials for specific groups of people	
Determining if those in most need of intervention were recruited	
In training sessions for new programs	
In training sessions for programs you have previously delivered	
Training others to deliver programming	
Traveling related to Extension program delivery	

Task	Average Time (per week)
Traveling related to Extension program training	
Delivering programs	
Developing and/or refining program materials	
Ensuring the program is delivered as intended (e.g., complete a checklist at the end of a program session)	
Maintaining partnerships for program delivery (e.g., attending community forums, networking, meetings)	
Evaluating the program	
Time spent working on adapting the program for future iterations	
Other (Please specify)	

2. How do you use technology professionally and personally?
(Please check all that apply).

☐Smartphone
☐Tablet (iPad, Note)
☐Laptop
☐Desktop
☐Social Media
☐Electronic Reader
☐Other __________

3. Please indicate your level of agreement/disagreement with the following statements where 1 = completely disagree and 5 = completely agree.

When I decide to deliver a program I value whether

	Completely Disagree<br>1	2	3	4	Completely Agree<br>5
1. This program helps people start being more active and "stick with it" even after the program is over.	1	2	3	4	5
2. This program fits the mission of University of Wyoming Extension.	1	2	3	4	5
3. This program will attract more residents in my area than other NFS programs.	1	2	3	4	5

	Completely Disagree 1	2	3	4	Completely Agree 5
4. This program would need to be adapted specifically to work within University of Wyoming Extension.	1	2	3	4	5
5. I could recruit a strong volunteer base using this program.	1	2	3	4	5
6. I could easily solicit support from local community organizations.	1	2	3	4	5
7. Other Extension Educators will help implement the program.	1	2	3	4	5
8. The program can be maintained/sustained easily for longer than one year without special funding.	1	2	3	4	5
9. The program will receive good public relations opportunities and visibility for me and Extension.	1	2	3	4	5
10. The program will become a Statewide "branded" program.	1	2	3	4	5
11. UWE will provide ongoing financial support for the program.	1	2	3	4	5

FitEx is an 8-week state-wide walking program based on group dynamics principles of goal setting, distinctiveness, social support, and accountability. In this program, teams of 6 work together to ensure that each member is achieving the physical activity guidelines of 150 minutes of moderate intensity physical activity per week.

Those who engage in the program improve their physical activity and fruit and vegetable consumption. After attending an in-person training, >90% of eligible health educators intended to deliver FitEx.

4. Would you be interested in receiving training on the delivery and evaluation of the FitEx program?
☐1 Yes ☐2 No

5. Please select all that apply. Would you be willing to receive this training:
__ In person
__ Webinar

6. What is your level of comfort delivering physical activity programs to older adults?

Very Uncomfortable — Very Comfortable

1 2 3 4 5

L.I.F.T., Lifelong Improvements through Fitness Together, is an 8-week, team-building, physical activity and nutrition program for older adults. Agents demonstrate exercises to promote balance, flexibility, and strength training that may improve the functional fitness of older adults and their ability to live comfortably, independent longer. The program will meet 2x/week allowing at least a day between sessions. Agents interested in delivering L.I.F.T. within their community will receive training on performing the functional fitness assessment with participants, the 8 full-body exercises completed throughout the program, and a thorough breakdown of the program manual in its entirety. This enables the agents to lead the program with confidence, ease, and familiarity.

7. Would you be interested in training on the delivery and evaluation of LIFT?
☐1 Yes ☐2 No

8. Please select all that apply. Would you be willing to receive this training:
__ In person
__ Webinar

PART C: Demographic and Health Information

1. Age: ____

2. Sex (check one): ☐1 Male ☐2 Female

3. Marital Status:
☐1 Single ☐2 Married ☐3 Widowed
☐4 Separated ☐5 Divorced ☐6 Living common-law or living with partner

4. Please indicate which of the following best describes you (Please Choose One).

☐1 White
☐2 Black or African American
☐3 Asian
☐4 American Indian/Alaskan Native
☐5 Native Hawaiian or Other Pacific Islander
☐6 Not sure
☐7 Other: ________

5. Please indicate which of the following best describes you (Please Choose One).

☐1 Hispanic or Latino
☐2 Not Hispanic or Latino
☐3 Not sure

6 General Health Status: In general, compared to other persons your age, how would you rate your health?

☐1 Poor ☐2 Fair ☐3 Good ☐4 Very good ☐5 Excellent

7. How confident are you that you can engage in moderate physical activities for 30 minutes for 5 or more days per week?

☐1 Not at all ☐2 Somewhat ☐3 Moderately ☐4 Very ☐5 Completely

8. Physical activity over the past week.
Considering the past 7-day period (last week), how many times did you do the following kinds of exercise for more than 15 minutes during your free time (write on each line the appropriate number). Only count exercise that was done during free time (i.e., not occupation or housework). Note that the main difference between the three categories is the intensity of the exercise. Please write the average frequency on the first line and the average duration on the second line.

	Times Per Week	Average Duration
a. STRENUOUS EXERCISE (HEART BEATS RAPIDLY, SWEATING)	__________	__________

(e.g., running, jogging, hockey, soccer, squash, cross country skiing, vigorous swimming, vigorous long distance bicycling, vigorous aerobic dance classes, heavy weight training)

b. MODERATE EXERCISE (NOT EXHAUSTING, LIGHT PERSPIRATION)	__________	__________

(e.g., fast walking, baseball, tennis, easy bicycling, volleyball badminton, easy swimming, alpine skiing, popular and folk dancing)

c. MILD EXERCISE (MINIMAL EFFORT, NO PERSPIRATION)	__________	__________

(e.g., easy walking, yoga, bowling)

9. Are you interested in participating in a phone interview that will elaborate on this line of questioning? You will be directed to a separate location to share your contact information.

a. Yes- taken to new survey to provide contact details.
(Name. Email. Phone number. County.)
b. No- Thank you for completing this survey.

Appendix B:
University of Wyoming Extension Educator Key Informant Interviews

Thank you for speaking with me today to participate in the University of Wyoming Extension (UWE) Educator key informant interviews. The purpose of key informant interviews is to collect information from community experts who have first-hand knowledge about the community and can provide insight about a topic. Today we want to discuss physical activity programming in UWE, covering topics ranging from curriculum to evaluation.

I am _______, and I am part of the research team. We will start by talking about your role as a participant in this study and obtaining informed consent. Then, I will start asking questions from my interview guide. My role as an interviewer is to present the topic areas, probe for any follow-up details we may need related to a specific response, and to keep track of time.

First I will read the consent form, allow time for any related questions, and collect signed consent for those of you who wish to continue with this focus group interview.

(Read consent form, collect signed consent forms. Resume [~5 minutes]).

There are no right or wrong answers, so please share your experience and thoughts as we continue.

I will begin recording now.

Please state your role in UWE and the county you are employed in.

(Allow interviewee to read their assigned participant number to both test for volume and 'record' voice recognition)

Thank you.

Physical activity programming (10-15 minutes)

What type of physical activity programming are you currently delivering in your counties?
    Probe(s): For how long, to whom?

What types of nutrition programs are you delivering that include a physical activity component?

What are some barriers for physical activity programming?
    Probe(s): knowledge, resources [space, equipment], interest from community members?

What are some things that make it easy to include physical activity programming in your counties?
    Probe(s): knowledge, resources [space, equipment], interest from community members?

Please describe the training you have as it relates to delivering physical activity programs.

Please describe your perceptions of support from your superiors to deliver physical activity programming.

What is your perception about the degree to which other Extension educators are delivering physical activity programming in UWE? What about other states?

How is your job performance evaluated?

How does physical activity programming relate to your job performance evaluation?

Please describe your thoughts on a "training/certification" for you to become more confident in delivering physical activity.

Personal

Please describe your confidence level that you will be able to successfully deliver physical activity programming.
Probes: What gives you that confidence, how has that changed, previous delivery, what makes unconfident
How prepared are you to deliver physical activity programming?
Probes: why, stages of change, current delivery
How do you determine which programs you deliver?
Probes: evidence, pragmatic vs. research based

As you may know, the national recommendation is for individuals to engage 30 minutes of moderate activity most days of the week.

Tell me how you feel about this recommendation.
Probe: how do you meet (or try to meet) these recommendations, feasibility

As you may know, the national recommendation is for individuals to include 2 or more days of full-body muscle strengthening in their physical activity routine.

Tell me how you feel about this recommendation.
Probe: how do you meet (or try to meet) these recommendations, feasibility

Wrap-Up (Remaining Time)

Is there anything else you would like to share with the research team at this time?

I am going to stop recording now.

Thank you for your participation in this key informant interview. Members of the research team will transcribe these sessions verbatim. We will then interpret the findings in order to conduct physical activity programming in UWE. If at any time during this process you wish to retract all or part of your statements, you may do so.

[Salutations]
